# Unmasking flawed exposure attribution in nicotine and cardiovascular risk

**DOI:** 10.1093/ehjopen/oeag059

**Published:** 2026-05-18

**Authors:** Red Thaddeus D Miguel, Isabella Steffensen

**Affiliations:** Thera-Business Inc., 515 Legget Dr, Suite 204, Kanata, ON K2K 3G4, Canada; Thera-Business Inc., 515 Legget Dr, Suite 204, Kanata, ON K2K 3G4, Canada

**This correspondence refers to ‘Nicotine and the cardiovascular system: unmasking a global public health threat.’, by T. Münzel *et al.*  https://doi.org/doi:10.1093/eurheartj/ehaf1010.**

**The authors' response is available at https://doi.org/10.1093/ehjopen/oeag065.**

We read with interest the publication by Münzel and colleagues.^1^ While the report addresses an important topic, its framing and inference exceed the supporting evidence and are already being interpreted as showing uniform cardiovascular harm from nicotine across products.

First, the manuscript advances cross-product conclusions by treating nicotine as a transferable causal driver across cigarettes, e-cigarettes, heated tobacco, waterpipes, and oral nicotine products. Within this framing, it also links nicotine-related products to ‘millions of deaths’ using smoking mortality estimates, without presenting corresponding mortality estimates for non-combusted products. This conflates a shared constituent with a shared exposure. Nicotine is the same molecule across products; what differs in clinically relevant ways is the exposure matrix that delivers it, including route of administration, nicotine pharmacokinetics, and the non-nicotine emissions and co-exposures that determine toxicologic dose. Accordingly, nicotine content is not a reliable proxy for nicotine uptake, and nicotine uptake alone does not capture cardiovascular hazard when co-delivered toxicants and inhaled particulate exposures vary by product. Framing these products as different ‘forms’ of nicotine collapses distinct exposure matrices and risks transferring the known cardiovascular burden of cigarette smoking to products whose toxicologic profiles have not been shown to produce comparable outcomes. Cross-product claims should be tied to evidence separating nicotine effects from emissions-driven risk.

The authors’ discussion of nicotine replacement therapy as low risk in clinical use and outweighed by cessation benefits drives the point home regarding what question should be at issue: whether nicotine, independent of product-specific co-exposures, increases cardiovascular events, in which populations, and relative to what comparator. We conducted a systematic review directly on nicotine and diagnosed cardiovascular events.^2^ Across 42 studies comparing nicotine and non-nicotine exposure groups, we found no significant associations with arrhythmia, non-fatal myocardial infarction, non-fatal stroke, or cardiovascular death (moderate certainty).^2^ Despite addressing nicotine’s independent effect on cardiovascular outcomes, our review was not identified by Münzel and colleagues.

Second, the manuscript draws long-term clinical implications from acute pharmacologic effects and short-term biomarker changes. Acute changes indicate physiological responsiveness but do not establish sustained vascular injury or increased risk of myocardial infarction, stroke, or cardiovascular death. These end-points are not interchangeable. They lack validated linkage to clinical outcomes in this context and are highly sensitive to transient, non-specific physiological stimuli that do not reliably predict disease progression. If endothelial function measures are intended to inform regulation, the manuscript should specify a context of use and provide evidence of reproducibility and outcome-linked validation in the relevant exposure setting.

Third, the manuscript presents epidemiologic associations for e-cigarette use and cardiovascular events in a manner that invites causal interpretation without adequately addressing dominant sources of bias. In most datasets, e-cigarette use exposure is strongly entangled with current or prior cigarette smoking and dual use, and temporality is frequently uncertain. A consensus review should either confine causal statements to evidence that addresses these biases or explicitly grade certainty and state what cannot be concluded.

Finally, the article is presented as a consensus synthesis but does not provide the transparency expected of an evidence-graded review supporting high-certainty conclusions.^3^ The methods for identifying, selecting, and weighting evidence are not explicit, and the manuscript does not clearly stratify by mechanistic plausibility, harmfulness of short-term biomarkers, and severity of clinical outcomes when making causal and policy claims.

The issues we raise here reflect a pattern of inference that moves beyond evidence synthesis and into assumption. The claim that nicotine, irrespective of delivery system, is sufficient to cause clinically meaningful cardiovascular harm is simply not supported by the data brought forward. The thesis of the paper is constructed by extending smoking-attributable mortality to all nicotine-containing products, interpreting acute physiological effects as long-term clinically significant outcomes, and presenting associations from observational studies without accounting for persistent confounding by cigarette smoking status or temporality. Precision in exposure attribution is essential in this domain. Reviews, policy papers, and other documents that aim to synthesize evidence while treating fundamentally different products as equivalent based on shared nicotine content risk overstating certainty and obscuring the actual drivers of harm. Where the evidence does not establish equivalence, policy should not presume it. This pattern is not unique to nicotine or cardiovascular science for that matter. It reflects a broader challenge in public health where precaution, when not precisely tied to the evidence, can blur rather than clarify risk communication.
